# Bacterial community structure of fresh and composted cattle manure revealed through 16S rRNA gene amplicon sequencing

**DOI:** 10.1128/mra.01092-24

**Published:** 2025-03-25

**Authors:** Munirah Tharek, Noriha Mat Amin

**Affiliations:** 1 Soil Science Water and Fertilizer Research Centre, Malaysian Agricultural Research and Development Institute (MARDI), Selangor, Malaysia; 2 Biotechnology and Nanotechnology Research Centre, Malaysian Agricultural Research and Development (MARDI), Selangor, Malaysia; Montana State University, Bozemana, Montana, USA

**Keywords:** fresh cattle manure, composted cattle manure

## Abstract

Data on the 16S rRNA gene amplicon sequencing from fresh and composted cattle manure are reported. The taxonomic distribution at the phylum level revealed Firmicutes as the most dominant phyla in fresh cattle manure, whereas Proteobacteria and Bacteroidota were the major phyla identified in composted cattle manure.

## ANNOUNCEMENT

Cattle manure is generated in significant quantities daily (1, 2). Livestock producers manage the manure through composting to reduce its volume and odor. Composting methods are widely used to convert manure into nutrient-rich organic fertilizers. This highly marketable composted manure is also applied to improve soil quality and productivity (3). However, ensuring proper composting practices can reduce the risk of microbial contamination, as manure contains a broad range of microbes, including pathogens (3, 4). To investigate the taxonomic differences in bacterial community structure between fresh and composted cattle manure, we conducted 16S rRNA gene amplicon sequencing. The results highlight the importance of using composted cattle manure and avoiding the direct application of fresh manure to mitigate potential health and environmental risks.

In December 2023, fresh and composted cattle manure were collected from the Malaysian Agricultural Research and Development Institute (MARDI) Research Station Kluang, Johor, Malaysia (latitude 1.954356°N, longitude 103.360800°E). Total DNA was extracted from 250 mg of fresh and composted cattle manure using the DNeasy PowerSoil Pro Kit (QIAGEN, Germany) and pooled. The integrity, purity, and concentration of the DNA were determined by observation through 1% Tris-acetate-EDTA (TAE) gel electrophoresis and analyzed at absorbance ratios of A260/280 and A260/230 using a spectrophotometer (Implen NanoPhotometer N60/N50) and fluorometric quantification using the iQuant Broad Range dsDNA Quantification kit. The purified DNA was amplified using locus-specific sequence primers as follows: 16S V3–V4 (forward overhang: 5′ TCGTCG-GCAGCGTCAGATGTGTATAAGAGACAG‐[CCTACGGGNGGCWGCAG]; reverse overhang: 5′ GTCTCGTGGGCTCGGAGATGTGTATAAGAGACAG‐[GACTACHVGGGTATCTAATCC]). All the PCR reactions were carried out with REDiant 2× PCR Master Mix (1st BASE). The first part of library construction involved the amplification of the 16S rRNA gene of the selected regions using locus-specific sequence primers. PCR reactions were performed with KOD Multi & Epi (Toyobo, Japan) (https://www.toyobo-global.com/seihin/xr/lifescience/support/manual/KME-101.pdf). The second part of library construction involved the attachment of dual indices to the amplicon PCR using Illumina Nextera XT Index Kit v2 following the manufacturer’s protocols. The quality of the libraries was measured using the Agilent Bioanalyzer 2100 System by Agilent DNA 1000 Kit and fluorometric quantification by Helixyte Green Quantifying Reagent. The libraries were normalized and pooled according to the protocol recommended by Illumina, followed by sequencing using the MiSeq platform with 300 bp paired-end reads. Raw data were clustered into amplicon sequence variants (ASVs) using DADA2 V1.18 (5) and aligned against the SILVA v132 database. Reads generated from fresh and composted cattle manure through the 16S rRNA gene amplicon sequencing are exhibited in Table 1.

**TABLE 1:** Sample information for 16S rRNA amplicon sequencing reads from fresh and composted cattle manure

Sample	Fresh cattle manure	Composted cattle manure
Input/raw	158,151	160,983
Filtered	49,445	40,372
Denoised F	48,967	39,917
Denoised R	49,125	40,036
Merged	43,975	36,013
Non-chimera	43,413	34,701
Sequence length distribution	35–301	35–301
Phred quality score (Q30)	23,364	30,595
NCBI accession	SRX25605173	SRX25605174

**Fig 1:**
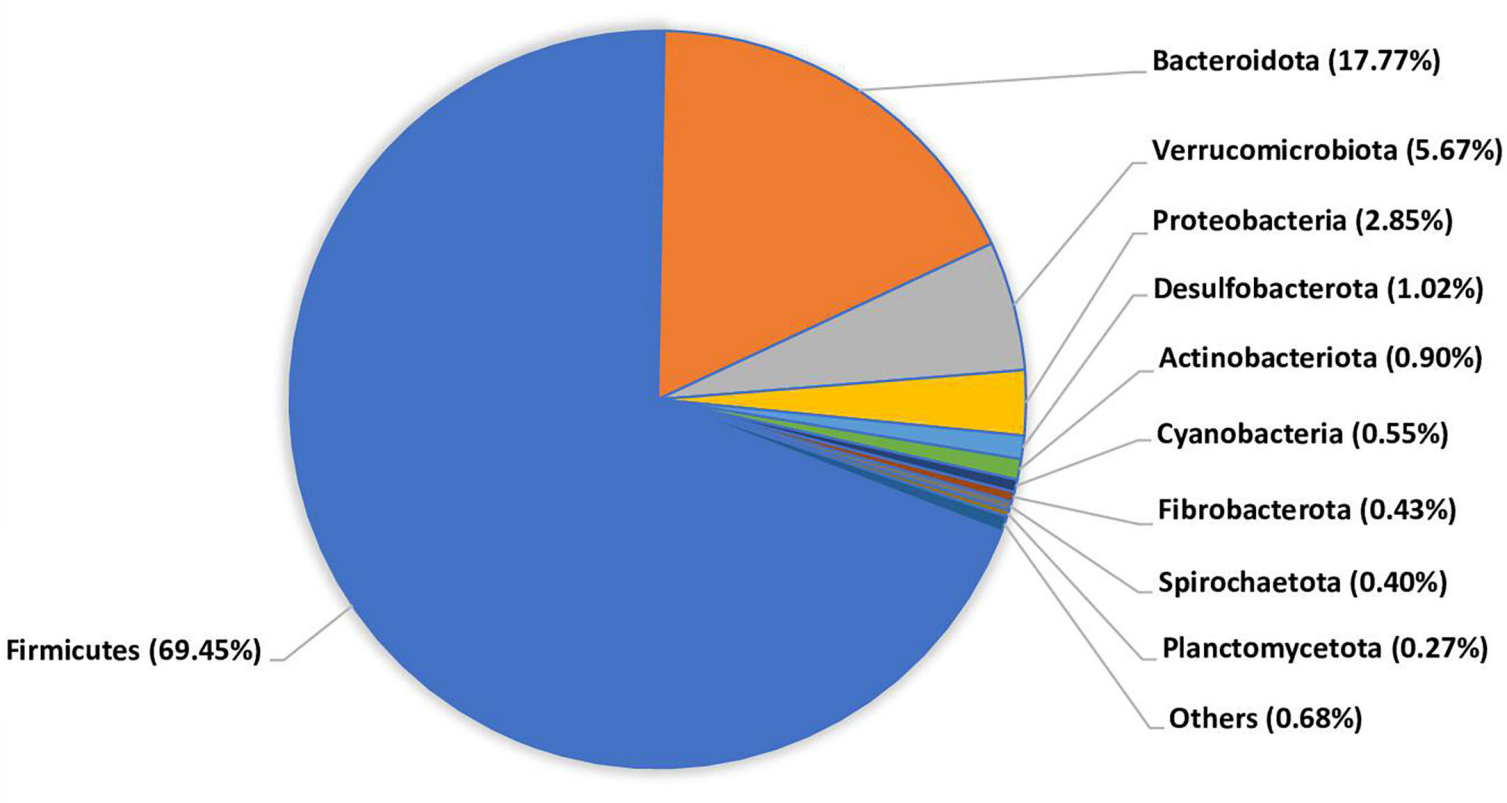
Percentage of key bacterial composition from fresh cattle manure.

Analysis at the phylum level revealed Firmicutes (69.45%) as the most abundant phyla in fresh cattle manure (Fig. 1), which is also known as the dominant bacteria phyla in cattle gut (6). While the major taxonomic groups in the composted cattle manure were Proteobacteria (26.88%) and Bacteroidota (13.20%) (https://doi.org/10.6084/m9.figshare.27166155.v1). These groups consist of bacteria involved in organic matter decomposition.

## Data Availability

The 16S rRNA gene amplicon sequencing data from this study have been deposited in the National Center for Biotechnology Information (NCBI) database under the Sequence Read Archive (SRA) accession numbers (SRX25605173 and SRX25605174) and the BioProject accession number PRJNA1142675.
